# Accelerated and intensified manufacturing of an adenovirus‐vectored vaccine to enable rapid outbreak response

**DOI:** 10.1002/bit.28553

**Published:** 2023-09-25

**Authors:** Carina C. D. Joe, Rameswara R. Segireddy, Cathy Oliveira, Adam Berg, Yuanyuan Li, Dimitrios Doultsinos, Steffi Scholze, Asma Ahmad, Piergiuseppe Nestola, Julia Niemann, Alexander D. Douglas

**Affiliations:** ^1^ Jenner Institute, Nuffield Department of Medicine University of Oxford Oxford UK; ^2^ Clinical Biomanufacturing Facility, Nuffield Department of Medicine University of Oxford Oxford UK; ^3^ Nuffield Department of Surgical Sciences John Radcliffe Hospital, University of Oxford Oxford UK; ^4^ Sartorius Stedim Biotech GmbH Goettingen Germany; ^5^ Repligen Corporation Waltham Massachusetts USA

**Keywords:** adenovirus, emergency response, outbreak response, pandemic response, perfusion, viral‐vector vaccine

## Abstract

The Coalition for Epidemic Preparedness Innovations' “100‐day moonshot” aspires to launch a new vaccine within 100 days of pathogen identification, followed by large‐scale vaccine availability within the “second hundred days.” Here, we describe work to optimize adenoviral vector manufacturing for rapid response, by minimizing time to clinical trial and first large‐scale supply, and maximizing output from the available manufacturing footprint. We describe a rapid virus seed expansion workflow that allows vaccine release to clinical trials within 60 days of antigen sequence identification, followed by vaccine release from globally distributed sites within a further 40 days. We also describe a perfusion‐based upstream production process, designed to maximize output while retaining simplicity and suitability for existing manufacturing facilities. This improves upstream volumetric productivity of ChAdOx1 nCoV‐19 by approximately fourfold and remains compatible with the existing downstream process, yielding drug substance sufficient for 10,000 doses from each liter of bioreactor capacity. This accelerated manufacturing process, along with other advantages such as thermal stability, supports the ongoing value of adenovirus‐vectored vaccines as a rapidly adaptable and deployable platform for emergency response.

## 1 INTRODUCTION

Enabling launch of a new vaccine within 100 days of pathogen identification has become a key objective of global vaccine research and development, having been stated as a central goal by the Coalition for Epidemic Preparedness Innovations and Pandemic Preparedness Partnership (CEPI, 2021). Achieving this requires vaccine platform technologies with known safety, immunogenicity, and manufacturing characteristics, such as messenger RNA (mRNA) and adenoviral vectors.

Strengths of adenovirus‐vectored vaccines include suitability for refrigerated storage, robust immunogenicity (including after a single dose), and accessible manufacturing technology. These features make the platform particularly attractive in low‐ and middle‐income countries. More than three billion doses of the “Oxford/AstraZeneca” adenovirus‐vectored COVID‐19 vaccine ChAdOx1 nCoV‐19 (AZD1222, Vaxzevria) were supplied in 2021–2022. The product has been used in 177 countries worldwide and was estimated to have saved around six million lives in 2021, more than any other COVID‐19 vaccine (Airfinity, 2022; AstraZeneca, 2021; Our World in Data, 2021). This was enabled by the rapid transfer of the platform manufacturing process to multiple production facilities, resulting in manufacturing having been distributed across 12 countries on five continents (Joe et al., 2021). Despite this success, manufacturing speed remains a perceived limitation of adenoviral vectors compared with other vaccine platforms, including mRNA (Hogan & Pardi, 2022; Kis et al., 2022).

Here, we describe work seeking to optimize each of three measures of a vaccine platform technology's suitability for emergency response to emerging pathogens and variants: time from pathogen sequence identification to supply of vaccine to clinical trial, time to first large‐scale product release, and time to release of one billion doses.

The two activities required for adenovirus manufacturing “start‐up” are the expansion of host cells and the generation of virus seeds with which to infect those cells (assuming that facilities, equipment, staff, and materials are already in place). These activities can take place in parallel and the latter is slower, making Good Manufacturing Practice (GMP)‐compliant virus seed generation the limiting factor for initiation of both trial‐scale and large‐scale manufacturing. Virus seed generation requires the synthesis of DNA encoding the antigen, insertion of this transgene into the adenoviral genomic construct, transfection of the genomic construct into producer cells to “rescue” the virus, limiting dilution to isolate clonal virus, and serial amplification. GMP requires time‐consuming quality‐control assays at multiple points in the seed‐production process, but the probability of failure is low, in our experience, for the most time‐consuming assays (detection of adventitious microbes and confirmation of genetic stability). Seed generation time can therefore be reduced by proceeding “at financial risk” to subsequent steps, in advance of assay results, provided that all required results will be available before vaccine release.

Following manufacturing start‐up, the ramp‐up capacity is constrained by the volumetric productivity of the manufacturing process and the facility cycle time (interval between batches). These factors determine the number of doses which can be produced each month per liter of bioreactor capacity, or per square meter of facility footprint.

Worldwide supply of ChAdOx1 nCoV‐19 used a process built upon a method that we developed before the COVID‐19 pandemic to enable rapid responses to emerging pathogen outbreaks (Fedosyuk et al., 2019; Joe et al., 2021). This was specifically designed to be quick and straightforward to adopt for manufacturing of any adenovirus‐vectored vaccine in any suitable facility, including those in low‐ and middle‐income countries. Use of a GMP‐compliant bank of suspension T‐REx‐293 cells (ThermoFisher) enables repression of transgene expression during production, which overcomes the potential problem of viral growth impairment by the transgene product. Only single‐use, off‐the‐shelf components are used throughout manufacturing, including both the upstream fed‐batch stirred‐tank bioreactor process and the downstream tangential‐flow filtration (TFF) and membrane‐based anion‐exchange chromatography process.

The volumetric productivity of the commercial ChAdOx1 nCoV‐19 production process is approximately 1.5 × 10^11^ viral particles (VPs) of drug substance (DS) per mL of bioreactor culture, or approximately 2000 final doses/L (Joe et al., 2021). Producing a final dose of 5 × 10^10^ VP requires upstream production of approximately 1.5 × 10^11^ VP because of losses during purification of DS (approximately 50% loss), fill‐finish, and extraction from vials (a further 33% of the DS, approximately), but relatively little scope exists for improving the efficiency of these stages. Major output improvement thus requires improved upstream volumetric productivity. Volumetric productivity (VP/mL) is the viable cell density (cells/mL) multiplied by the cell‐specific productivity (VP/cell) during viral vector replication in the producer cell line (i.e., volumetric productivity = viable cell density × cell‐specific productivity).

The “cell density effect” is the principal factor limiting the volumetric productivity of adenoviral vectors (Dormond et al., 2009; Nadeau & Kamen, 2003). Cell densities above 10^7^ viable cells/mL are readily achievable in fed‐batch growth of uninfected producer cell lines in stirred‐tank bioreactors (Petiot et al., 2015). Unfortunately, cell‐specific productivity of adenoviral vectors drops very sharply at quite modest cell densities, above approximately 1–2 × 10^6^ viable cells/mL at infection (corresponding to somewhat higher peak viable cell density after infection) (Shen et al., 2010). As a result, increasing the cell density beyond this modest level does not improve the volumetric productivity of adenoviral vectors (Dormond et al., 2009; Ferreira et al., 2009; Nadeau & Kamen, 2003). High cell‐specific productivity has an important additional benefit on top of its effect upon volumetric productivity, in that a high ratio of active product to cell‐derived impurities facilitates downstream processing.

In our fed‐batch ChAdOx1 nCoV‐19 manufacturing process at large scale, we have observed cell‐specific productivity of approximately 3–5 × 10^4^ VP/cell, with a peak viable cell density in the region of 4–6 × 10^6^ cells/mL (Joe et al., 2021). This cell‐specific productivity value is in line with previously published results with ChAdOx1 and other species E simian adenoviral vectors and with human adenoviral vectors (Fedosyuk et al., 2019; Nadeau & Kamen, 2003). The combination of cells, antigen repression, vector engineering, medium, and feed allows this cell‐specific productivity to be maintained at somewhat higher cell densities than previously reported, resulting in increased volumetric productivity. Nonetheless, overcoming the cell density effect remains a central challenge in boosting ChAdOx1 nCoV‐19 manufacturing output (Joe et al., 2021).

The physiological basis of the cell density effect is incompletely understood, but an inhibitory effect of cellular waste products upon viral replication is a likely contributor. Production of adenoviral vectors is fundamentally a batch process because a single cycle of viral replication lyses the producer cells over a time frame of 24–72 h (Nadeau & Kamen, 2003). This contrasts with the production of other biological products like monoclonal antibodies, in which constitutive expression enables continuous processes. Although viral replication rapidly increases cellular energy demand (Maranga et al., 2005), nutrient supplementation with glucose, vitamins, amino acids, or nucleotides does not raise the cell density effect barrier beyond about 1–2 × 10^6^ viable cells/mL at infection (Dormond et al., 2009; Maranga et al., 2005; Shen et al., 2010). Cell‐specific productivity is impaired by metabolic waste products, such as ammonia (from glutamine metabolism) and lactate (from anaerobic glucose metabolism) (Ferreira et al., 2005, 2007; Maranga et al., 2005). Medium exchange by centrifugation or TFF before or during the brief period of adenoviral vector replication in the producer cells can both replenish nutrients and remove waste products. Alternating tangential‐flow filtration (ATF) and levitating magnetic pump head systems can accomplish this with low shear forces, an important consideration for fragile virus‐infected producer cells. In contrast to nutrient supplementation, medium exchange by perfusion is reportedly effective in raising the cell density effect barrier in processes using species B, C, and D human adenoviral vectors (Cortin et al., 2004; Galvez et al., 2012; Henry et al., 2004; Yuk et al., 2004). As compared with fed‐batch processes, however, perfusion requires additional equipment and the use and handling of substantial quantities of additional medium. It is perceived by some as being a complex operation that some manufacturing sites may find difficult to implement.

Here, we demonstrate that medium exchange using ATF can raise the cell density effect barrier for the production of ChAdOx1 nCov‐19. Using the simplest possible perfusion conditions, we show that increasing viable cell density at infection (VCDI) to 6 × 10^6^ cells/mL improves volumetric productivity by approximately fourfold, with no loss of cell‐specific productivity.

We also provide a workflow for rapid production of working virus seed to support our new process at global scale, enabling large‐scale vaccine release within 100 days from pathogen sequence identification.

Historically, in contrast with manufacturing of some other virus‐based vaccines, most adenovirus production methods have involved infection of all producer cells in a “single hit” with a multiplicity of infection (MOI) above 1 infectious unit (IU)/cell, followed by harvest approximately 2–3 days later, after a single viral lifecycle (Fedosyuk et al., 2019; Luitjens & Van Herk, 2016; Nadeau & Kamen, 2003). Our previous work has used such “high‐MOI” single‐lifecycle processes throughout seed amplification. After limiting dilution to isolate clonal virus, we have typically used adherent cells to perform approximately four amplification steps in a pre‐GMP laboratory. At each stage, virus is recovered by freeze–thaw‐mediated cell lysis and the infectious titer in the lysate is measured to determine the MOI to use for the following step. A large “safety factor” is typically allowed at each stage (i.e., oversizing the culture to allow for the possibility of poor output). GMP master virus seed (MVS) and working virus seed production then proceeds similarly, but in stirred‐tank bioreactors and using more efficient detergent‐mediated lysis. It would be challenging to produce sufficient working virus seed using this approach to meet the needs of a global campaign in which MOI is above 1 IU/cell in the manufacturing process. This was one of the factors which motivated development of the “two viral lifecycle” process used for commercial production of ChAdOx1 nCoV‐19 (Joe et al., 2021). Use of an MOI considerably lower than 1 IU/cell, in conjunction with an extended postinfection culture period of 5–6 days, allows an additional cycle of viral amplification to occur within the production bioreactor, and substantially reduces the requirement for the input of working virus seed (Joe et al., 2021). To our knowledge (and perhaps surprisingly, given the existing use of similar approaches for other viruses) this approach had not previously been used in the adenovirus field.

To summarize, we have previously used high‐MOI processes for seed generation, and a low‐MOI process for DS production. Here, we reversed the above approach, reasoning that the high viral “amplification factor” achieved by a two‐cycle/low‐MOI culture would be ideal for generation of virus seed with the minimum number of amplification steps, and hence the minimum burden of testing between stages. By facilitating the production of much larger quantities of working virus seed than previously, this new approach also enables a high‐MOI production process. High‐MOI DS production minimizes time in the production bioreactor and, through synchronous infection, may improve product consistency (avoiding complexities arising from heterogeneity of timing of cell infection and death, such as accumulation of cell debris and potential loss of product into the perfusion permeate).

## 2 RESULTS

### 2.1 Rapid large‐scale virus seed supply

Rapid virus seed supply requires an upstream process which will rapidly amplify the virus, a downstream process which will recover it in a suitable form for use as seed, and supporting analytics.

**Figure 1:**
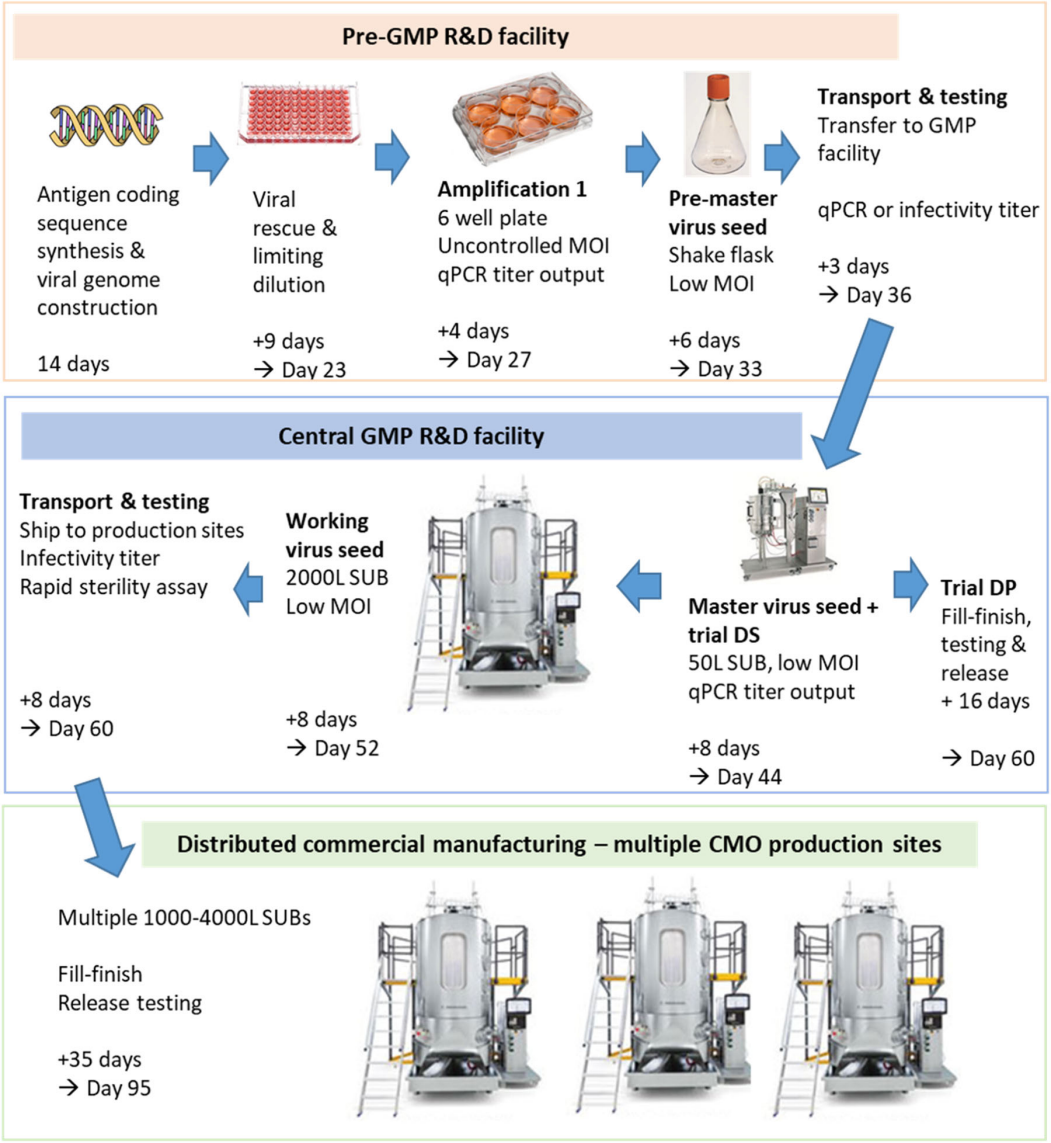
Rapid virus seed production enables early vaccine release. Detailed scheme for virus seed production, including anticipated timing of each step. This timing assumes the availability of facilities, equipment, materials, and staff. It further assumes cell expansion for MVS, WVS, and DS production occurring in parallel with preceding steps of virus seed generation, potentially making use of a “bleed and dilute” strategy to hold cells at 50–200 L volume and hence ensure immediate readiness for WVS/DS production upon seed availability. Finally, it assumes decisions to proceed “at financial risk” at all stages, ahead of the availability of any test results other than those stated, but with the completion of full testing before drug product release. CMO, contract manufacturing organization; DP, drug product; DS, drug substance; GMP, Good Manufacturing Practice; MOI, multiplicity of infection; MVS, master virus seed; qPCR, quantitative polymerase chain reaction; R&D, research and development; SUB, single‐use bioreactor; USP, upstream process; VPs, viral particles; WVS, working virus seed.

Because low‐MOI processes have now been extensively characterized in suspension cells, including the use of highly efficient detergent‐mediated (rather than freeze–thaw) lysis to recover virus, we sought to make the earliest possible transition to suspension cell culture. We estimated that a single uncontrolled‐MOI amplification step in adherent cells in a six‐well plate, followed by a low‐MOI suspension‐cell‐based amplification step in a 30‐mL shake flask would provide sufficient premaster seed to infect a 50‐L stirred‐tank bioreactor. This in turn could provide both ≥10,000 doses of clinical trial DS and adequate MVS to meet the needs of a global production campaign (Figure 1 and Table 1). With respect to a downstream process, we reasoned that incorporation of depth filter clarification, anion‐exchange chromatography (AEX), and 0.2 μm filtration, using appropriately scaled versions of the established downstream unit operations, would provide low bioburden virus seed (whether premaster, master or working seed) in a controlled buffer matrix without detergent.

**Table 1:** Performance of workflow for working virus seed production.

Step	Premaster virus seed	Master virus seed	Working virus seed
Nominal vessel volume (L)	0.25	50	2000
Culture working volume used for seed (L)	0.03	20^a^	1600
Cell density at infection (cells/mL)	0.8 × 10^6^		
Multiplicity of infection (VP/cell)^b^	6		
Required input (VP)	1.4 × 10^8^	1.9 × 10^11^	7.7 × 10^12^
Required input as proportion of previous output	–	0.194	0.012
Predicted usable output (VP)^c^	9.9 × 10^11^	6.6 × 10^14^	5.3 × 10^16^
Observed usable output (VP)	4.5 × 10^12^	2.0 × 10^15^ d	1.6 × 10^17^ d

Abbreviations: IU, infectious unit; pPCR, quantitative polymerase chain reaction; VPs, viral particles.

^a^ On the basis of the assumed total working volume of 40 L at master virus seed stage, with half of this used to provide drug substance for a clinical trial.

^b^ On the basis of quantitative PCR; corresponds to 0.1 IU/cell (plausible range, 0.03–0.20 IU/cell) assuming a typical particle:infectivity ratio.

^c^ Assumes 1.0 × 10^11^ VP/mL upstream productivity and 33% recovery by depth filtration and anion‐exchange chromatography.

^d^ Values for master virus seed and working virus seed are extrapolated from 3.0 × 10^14^ VP (measured by qPCR, as the mean of triplicate samples each assayed in triplicate wells on a single plate) obtained in a 3‐L working‐volume scaled‐down model (i.e., 1.0 × 10^11^ of seed recovered per milliliter of culture).

Virus grown in cells from a previously tested master cell bank by skilled operators is highly unlikely to be contaminated with bacteria or mycoplasma. Immediate transfer of such working virus seed to production facilities, in advance of pharmacopoeial sterility and mycoplasma test results, may be accepted as posing a modest and purely financial risk (rather than patient safety risk). This risk could be further mitigated by the use of rapid sterility and mycoplasma assays. To further streamline testing between amplification steps, we reasoned that calculation of MOI on the basis of genome‐containing VPs would be an accurate enough estimate of infectious titer to achieve reasonable productivity during amplification, given that particle:infectivity ratios are rarely outside the range 30–300 (for the avoidance of doubt, and as discussed in Section 4, throughout this manuscript the term “viral particle” and the abbreviation “VP” refer to genome‐containing particles). This approach would enable a rapid quantitative polymerase chain reaction (qPCR) assay of the input virus, rather than a time‐consuming infectivity assay.

To test this workflow, we performed a simulated low‐MOI premaster working virus seed amplification step at 30 mL scale, using syringe‐driven filtration through a 23‐cm^2^ depth filter and 3 mL AEX membrane. After infection at a qPCR‐determined MOI of 6 VP/cell, we obtained an output of 4.5 × 10^12^ VP, or greater than 20 times that required in our workflow to infect a 50‐L bioreactor for the production of working virus seed. We then used a 3‐L culture volume in a stirred‐tank bioreactor as a model of both 50‐L‐scale MVS production and 2000‐L working virus seed production. This was infected at a qPCR‐determined MOI of 6 VP/cell, using an aliquot of the simulated premaster seed which had been stored frozen. The culture was harvested, clarified, and subjected to AEX as previously described for our low‐MOI fed‐batch process (Joe et al., 2021). This provided an output of 1.0 × 10^11^ VP of AEX‐purified seed per mL of culture (Table 1). Based upon this productivity, allocation of 20 L of MVS would be sufficient for around 200 WVS batches (each of 2000 L), more than any program would be likely to require. We did not assess the possibility that the use of qPCR rather than infectivity for the calculation of MOI during seed production could increase yield variability, but the large amount of excess seed produced in this experiment suggests that even very substantial yield variability would not threaten a program.

Using assay turnaround times based upon our previous experience, we estimate that this workflow could allow 10,000 doses of vaccine to be released to clinical trial within 60 days of antigen sequencing. Working virus seed could be provided to globally distributed large‐scale manufacturing sites at the same time. This in turn could permit the release of a fully tested vaccine at a large scale after a further 35 days, that is, less than 100 days in total from pathogen identification (Figure 1).

### 2.2 Cell density effect in a model fed‐batch process

Beyond the production of the first clinical trial batch, the speed at which additional doses can be made available is closely related to process productivity. We therefore next looked to improve volumetric productivity, to increase the output of each liter of installed bioreactor capacity in each batch.

**Figure 2:**
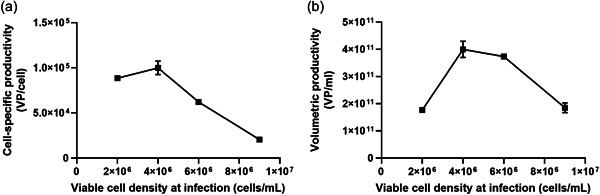
Limitation of productivity by the “cell density effect.” (a) Cell‐specific and (b) volumetric productivity of ChAdOx1 nCov‐19 over a range of viable cell densities at infection. An MOI of 5 was used, and flasks were harvested at 44 h. Data are given as median and range (if large enough to display) from duplicate shake flasks. Productivity values were based on viral particles measured by qPCR. Viable cell density was measured at infection. MOI, multiplicity of infection; qPCR, quantitative polymerase chain reaction; VPs, viral particles.

We initially sought to determine the effect of cell density in limiting the volumetric productivity of ChAdOx1 nCoV‐19 in T‐REx‐293 cells, using a scaled‐down fed‐batch process in shake flasks. Volumetric productivity was not improved by increasing cell density above 4 × 10^6^ cells/mL at infection, owing to declining cell‐specific productivity (Figure 2). Maximal volumetric productivity before downstream processing was approximately 4 × 10^11^ VP/mL.

### 2.3 High‐throughput assessment of medium exchange

**Figure 3:**
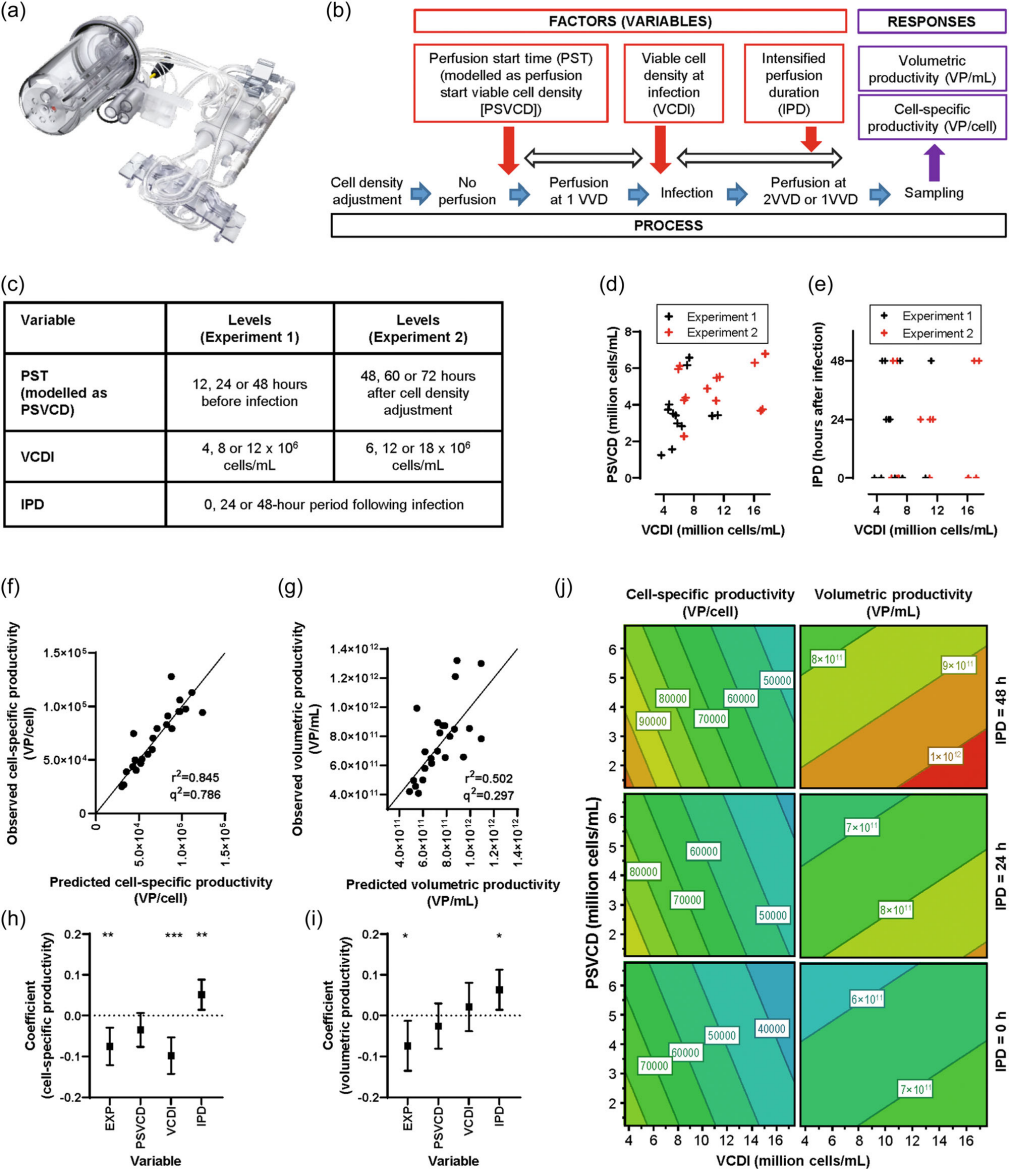
High‐throughput assessment of perfusion parameters on productivity. (a) Ambr250 perfusion bioreactor system, with perfusion unit for medium exchange via alternating tangential‐flow filtration. (b) Experimental design showing the three factors tested as independent variables and the two responses tested as dependent variables. (c) High, low, and center‐point levels for each variable in experiments 1 and 2. (d, e) Multivariate regression model parameter space for the three variables across each bioreactor in experiment 1 (black) and experiment 2 (red); note that PST is converted to PSVCD for modeling because of the different way in which PST was triggered in the two experiments. (f, g) Scatter plots of observed versus predicted values assessing model goodness of fit for the two productivity response variables; diagonals show lines of identity. (h, i) Modeled coefficients for the four factors for each of the two productivity response variables; bars show 95% confidence intervals. (j) Contour plots of modeled productivity, with the “experiment” factor held constant at the mean, illustrating relative effects on the responses across the ranges of the variables but not representing predictions; modeled values are likely to be particularly unreliable beyond the experimental design space. *p < 0.05, **p < 0.01, ***p < 0.001 (*t* test vs. null hypothesis of parameter coefficient being zero). EXP, experiment; IPD, intensified perfusion duration; PST, perfusion start time; PSVCD, perfusion starting at a viable cell density; VCDI, viable cell density at infection; VPs, viral particles; VVD, vessel volume(s) per day.

We next sought to explore conditions under which a perfusion‐based process might enhance productivity while adding as little complexity as possible to the process. We used the Ambr250 High‐Throughput Perfusion multiparallel bioreactor system (Ambr250 HT perfusion; Sartorius) with ATF‐mode medium‐exchange units (Figure 3a) to assess the effect of three controlled factors on volumetric and cell‐specific productivity: VCDI, perfusion start time (PST), and duration of intensified perfusion after infection (Figure 3b). High and low levels for each factor were assessed in a 2 × 2 × 2 factorial design, with additional center points, in two independent experiments (Figure 3c; Supporting Information Table 1). Across both experiments, this approach provided a parameter space encompassing: perfusion starting at a viable cell density (PSVCD) in the range of approximately 1–7 × 10^6^ cells/mL, a VCDI in the range of 4–18 × 10^6^ cells/mL, and a duration of intensified perfusion after infection of 0, 24, or 48 h (Figure 3d–e).

Following regression modeling, scatter plots of actual versus predicted values showed acceptable model fits, with *r* ^2^ values of 0.845 for cell‐specific productivity and 0.502 for volumetric productivity (Figure 3f–g). Observed data for each bioreactor are shown in Supporting Information Figure 1. Sensitivity analyses indicated that the estimated factor coefficients were robust to a variety of changes in the approach to analysis, including analysis of the first and second experiments in isolation (Supporting Information Fig. 2).

The regression model indicated that longer intensified perfusion duration (IPD) significantly improved both cell‐specific productivity (*p* < 0.01) and volumetric productivity (*p* < 0.05) (Figure 3h–i). Lower VCDI significantly improved cell‐specific productivity (*p* < 0.001), but not volumetric productivity. The effect of perfusion start viable cell density was not statistically significant, but trended towards increased productivity with earlier perfusion start.

Productivity metrics were also statistically significantly influenced by whether the bioreactor was part of the first or second experiment. Cell‐specific productivity was lower in the second experiment, in which mean values of two of the controlled factors (VCDI and PSVCD) were both higher, by design. Small changes to the culture conditions had also been made for the second experiment, in anticipation of the higher VCDI (reduction in volumes in the shake flasks during the seed train, with the intention of reducing aggregation, and an additional feed before the start of perfusion). It is not possible to determine from our data which of these changes were responsible for the lower cell‐specific productivity in that experiment. Although further characterization of the impact of these parameters would be desirable as part of the development of a robust final process, we did not consider the difference between experiments to be problematic for the interpretation of our data. Our aim was to gather information on the influence of each factor (which was consistent between experiments, as per the sensitivity analyses), rather than to predict absolute values of the responses in a hypothetical future experiment.

Under the optimal modeled conditions, cell‐specific productivity before downstream processing was predicted to exceed 10^5^ VP/cell and volumetric productivity to exceed 10^12^ VP/mL (Figure 3j). The highest observed volumetric productivity was 1.3 × 10^12^ VP/mL, in two bioreactors in the first experiment, which had VCDI of 6 and 11 × 10^6^ cells/mL (Supporting Information Table 1). This was not associated with impaired cell‐specific productivity, which remained at approximately 1 × 10^5^ VP/cell in both these bioreactors. Volumetric and cell‐specific productivity was close to these maximum values in simplified center‐point‐like bioreactors in both experiments, in which perfusion was started early but was not intensified after infection (Supporting Information Table 1).

### 2.4 Perfusion‐based upstream process at 3 L scale

**Figure 4:**
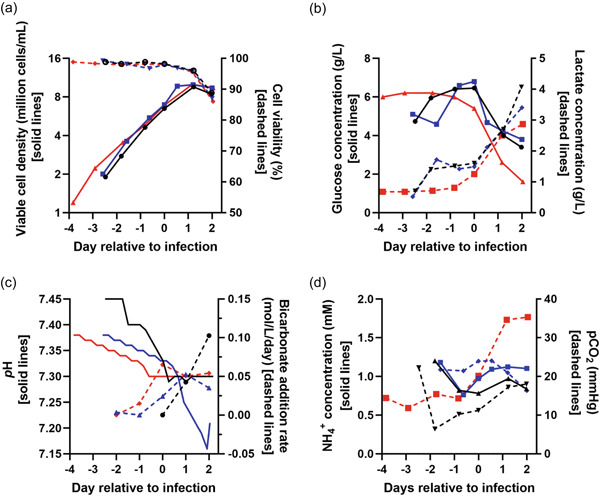
Perfusion‐based upstream process performance in three independent 3‐L‐scale production runs. Data from three independent production runs were conducted on separate occasions. Medium exchange via ATF perfusion started at 1 vessel volume/day at day –2 relative to infection and continued to day +2 relative to infection, when the bioreactors were harvested. (a) Viable cell density and cell viability, (b) glucose and lactate concentration, (c) pH and rate of bicarbonate addition, and (d) ammonia concentration and partial pressure of carbon dioxide (*p*CO_*2*_). See Table 2 for productivity data. Individual runs are represented as follows; experiments referred to as CJ74 in black, CJ78 in red, and CJ87 in blue. Parameters represented by solid and dashed lines are indicated on the left and right *Y*‐axes, respectively. ATF, alternating tangential‐flow filtration.

We next tested a new upstream process using ATF medium exchange in three independent accurately simulated production runs in 3 L single‐use bioreactors. Conditions were similar to those in the simplified center‐point‐like bioreactors in the multiparallel experiments, with early perfusion start but no intensification. Perfusion with 1 vessel volume/day starting 48 h before infection, at a viable cell density of approximately 3 × 10^6^ cells/mL, resulted in a VCDI of 6.5–7.0 × 10^6^ cells/mL across the three runs (Figure 4a and Table 2). Viable cell density peaked at approximately 1 × 10^7^ cells/mL and viability then declined as expected during viral replication.

**Table 2:** Productivity and performance of the improved perfusion‐based upstream process.

Parameter	CJ74	CJ79	CJ87
*Upstream process*			
Volumetric productivity (VP/mL of culture)	1.4 × 10^12^	1.6 × 10^12^	1.6 × 10^12^
Viable cell density at start of perfusion (cells/mL)	2.8 × 10^6^	3.6 × 10^6^	3.5 × 10^6^
Viable cell density at infection (cells/mL)	6.5 × 10^6^	7.0 × 10^6^	6.9 × 10^6^
Viable cell density at peak (cells/mL)	9.6 × 10^6^	1.0 × 10^7^	1.0 × 10^7^
Cell‐specific productivity (VP/cell) based on peak viable cell density	1.5 × 10^5^	1.6 × 10^5^	1.6 × 10^5^
*Purified bulk drug substance*			
Volumetric productivity (VP of DS/mL of upstream culture)	8.1 × 10^11^	8.3 × 10^11^	7.9 × 10^11^
Particle:infectivity ratio	35	42	77
Host‐cell protein (ng/mL)	33	61	79
*A*_260_:*A*_280_ ratio	1.17	1.18	1.15
Host‐cell DNA (ng/dose)	<0.1	<0.1	<0.1

*Note*: This table shows data from the three experiments reported in Figures 4 and 5. Further information about downstream process performance is provided in Supporting Information Table 2.

Abbreviations: DS, drug substance; VPs, viral particles.

Glucose concentrations decreased and lactate concentrations increased from the day of infection onwards (Figure 4b). Small ongoing reductions in pH were associated with increasing rates of bicarbonate addition from the day of infection onwards (Figure 4c), with little change in ammonia and carbon dioxide levels (Figure 4c). ATF filter transmembrane pressure remained stable between –0.8 and –0.9 bar throughout perfusion, indicating no significant fouling of the membrane.

Volumetric productivity before downstream processing was 1.4–1.6 × 10^12^ VP/mL across the three simulated production runs and cell‐specific productivity was 1.5–1.6 × 10^5^ VP/cell (Table 2).

### 2.5 Compatibility with existing downstream process

Many modern adenovirus downstream processes combine AEX and removal of low molecular weight impurities by TFF, with typical recoveries c. 50% (European Medicines Agency, 2020; Fedosyuk et al., 2019; Joe et al., 2021; Vellinga et al., 2014). The commercial downstream process for ChAdOx1 nCoV‐19 was dispensed with TFF before AEX, reducing filter, equipment, and buffer requirements for large‐scale production (Joe et al., 2021).

**Figure 5:**
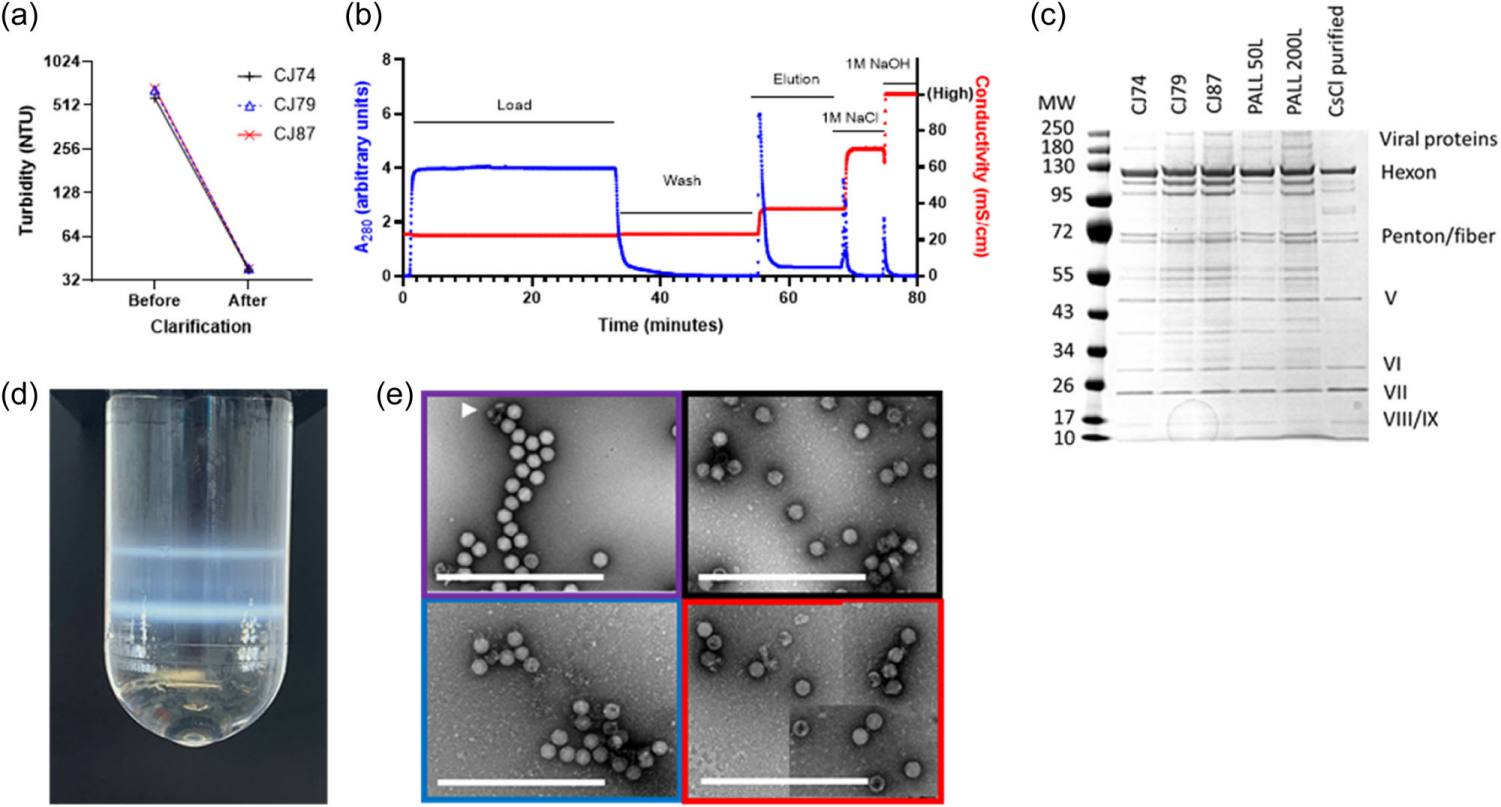
Downstream process performance and product characterization. (a) Turbidity before and after clarification of crude cell lysate from the three independent model production runs conducted on separate occasions: CJ74 in black, CJ78 in red, and CJ87 in blue. (b) Representative anion‐exchange chromatogram for purification of product from these three runs. Note that the maximal measurable conductivity was 100 mS/cm. Blue symbols indicate *A*_260_, red symbols indicate conductivity. (c) Silver‐stained denaturing and reducing sodium dodecyl sulfate polyacrylamide electrophoresis gel loaded with downstream processed material from the three model production plus two comparators from previously‐reported fed batch 50L and 200L runs performed by Pall Corporation 2021 and CsCl density gradient ultracentrifugation‐purified product. Note that all production samples contain additional bands, some of which will correspond to empty viral capsids compared with CsCl‐purified control, as expected for the chromatographic downstream process. (d) Representative analytical CsCl density gradient ultracentrifugation from one of the three model production runs showing lower band corresponding to genome‐containing viral particles and upper band corresponding to empty viral capsids. (e) Representative negative‐stain transmission electron micrographs of product from the three model production runs, color coded as in panel (a), together with CsCl ultracentrifugation‐purified comparator (purple). Note that empty capsids admit, and complete capsids exclude, the contrast agent. Arrowhead indicates artefactual fragments resulting from sample preparation. Scale bars indicate 1 µm. Red‐bordered image is a composite of three separate images (used to provide a view of a similar number of particles to those seen for other preparations, because this imaged preparation contained a lower viral particle concentration). Consistent with panel (c), background in the images of chromatographically prepared samples is believed to represent host cell and viral protein (not seen after CsCl ultracentrifugation, but similar to our experience of other chromatographically prepared samples). GMP, Good Manufacturing Practice; MW, molecular weight; NTU, nephelometric turbidity units.

To assess the compatibility of our new upstream perfusion‐based process with that existing downstream process, we purified the product from the three independent upstream runs using two versions of our previously published method (Joe et al., 2021). These comprised clarification by combined depth filtration and 0.2 µm filtration (Figure 5a), purification by AEX chromatography (Figure 5b), and formulation by TFF and 0.2 µm filtration. Product recovery after each step and filter loadings were within the expected ranges (Supporting Information Table 2). Addition of an extra TFF step after clarification in one run did not alter product recovery (Supporting Information Table 2).

Quality assays indicated that particle:infectivity ratio and host‐cell protein (HCP) and DNA content were within the acceptable ranges for all three runs (Table 2). The product contained the same viral capsid proteins as product derived from the conventional process (Figure 5c). The presence of an expected minor component of empty viral capsids, consistent with chromatographic purification and the observed *A*_260_:*A*_280_ ratios of 1.15–1.18, was confirmed by density gradient ultracentrifugation (Figure 5d) and electron microscopy (Figure 5e). Volumetric productivity after downstream processing was approximately 8 × 10^11^ VP of DS/mL of bioreactor culture, with very similar values across the three runs (Table 2), representing downstream efficiency of approximately 50%. This would be sufficient for >10,000 finished doses/L of bioreactor working volume.

This productivity implies that, despite the increased virus seed requirements of a high‐MOI process, WVS from a single 2000‐L bioreactor run (Table 1) could seed production of sufficient DS for >100 million finished doses. This calculation is based upon assumptions we believe to be cautious, that is, the output of 5.3 × 10^16^ VP/2000‐L WVS reactor, particle:infectivity ratio of 100, 33% wastage due to QC and aliquoting, and infection at 6 × 10^6^ cells/mL with an MOI of 5 IU/cell.

## 3 DISCUSSION

The SARS‐CoV‐2 genome sequence was published on January 10, 2020 (Holmes & Zhang, 2020). The adenovirus‐vectored vaccine ChAdOx1 nCoV‐19 was administered to the first volunteer in a clinical trial after 104 days (April 23, 2020) and administered for the first time outside a clinical trial after 360 days (January 4, 2021) (BBC News, 2021). Release of the billionth dose was announced after 566 days (July 29, 2021) (University of Oxford, 2021). The first mRNA vaccine (from Pfizer) and inactivated SARS‐CoV‐2 vaccine (from Sinovac) to release one billion doses did so about the same time (Our World in Data, 2021). The rates of progress of leading COVID‐19 vaccines to each of these milestones have been substantially faster than had been achieved for any previous novel vaccine.

We recently published a technoeconomic model of the process described here (Joe et al., 2023). This suggested that, if our current understanding had been available immediately at the time of SARS‐CoV‐2 sequence publication and production had proceeded as described here, one billion finished doses could have been available for release more than a year earlier, before the end of May 2020. This would have required significant investment “at financial risk,” and the availability of both DS and drug product production facilities, staff, equipment, and materials “on standby.” Translation of this manufacturing speed into rapid public health impact would require equally fast preclinical and clinical development (the same applies to other vaccine platforms). This may or may not be achievable for a completely new pathogen, but almost certainly is achievable for a new version of an existing vaccine in response to a new pathogen variant (e.g., SARS‐CoV‐2 or potentially influenza). Importantly, since the SARS‐CoV‐2 pandemic, the UK regulator (the Medicines and Healthcare Products Regulatory Agency) has advised that a Phase I trial of another ChAdOx1‐vectored vaccine could be authorized without a new GLP toxicology study, including in a nonemergency situation, by cross‐reference to previous data obtained with other ChAdOx1 vaccines (Sarah Gilbert, personal communication).

We expect a similar acceleration of manufacturing will also be possible for other vaccine platforms. The factor limiting the speed of vaccine availability may be the speed of early decision‐making. Willingness to invest “at financial risk” in the early stages of seed generation, facility preparation, and clinical trial preparation (long before it is even clear there is a public health need for any vaccine in response to a new and incompletely characterized outbreak) provides a global public good.

This work used a single vector (ChAdOx1 nCoV‐19). As we have found in previous work with a nonperfusion batch process, our expectation is that the use of a cell line which represses antigen production (such as HEK293 Trex, as used here) will render manufacturing independent of antigen, while modest changes in AEX buffers are sufficient for adaptation to non‐ChAdOx1 serotypes (Fedosyuk et al., 2019). Nonetheless, confidence in the applicability of this method as a true platform process will require confirmation of its applicability to adenovirus‐vectored vaccines using other serotypes and delivering other antigens.

For an adenovirus‐vectored vaccine, rapid seed generation remains a key determinant of time to trial‐scale and large‐scale vaccine supply. Because the seed generation and perfusion processes described here are independent of each other, and the seed generation method involves only modest changes to existing processes, we believe that the timelines outlined for trial‐scale and large‐scale supply could be achieved almost immediately. The main changes required are adoption (at premaster, master, and working virus seed stages) of low‐MOI two‐lifecycle processes, which are already well established for DS production. Analytical demonstration of the comparability of vaccine produced using different upstream processes for the initial trial batch and large‐scale production would also be required.

Speed of early manufacturing depends critically on the willingness of the GMP seed‐production facilities to accept incoming starting material before microbiological testing is completed. Such acceptance does not pose a risk to product quality (as the result would be available long before batch release) but does pose some business risk. This may be problematic, particularly in the early stages of an outbreak when the level of demand for a product will be uncertain. Use of rapid, culture‐independent nucleic‐acid‐based test methods would mitigate this risk.

Overall, we believe that each step of the seed‐production process is robust and that risks of assay failures are low. It would nonetheless seem prudent, on both public health and financial grounds, to invest additional effort in mitigating risks of failure (e.g., with a preplanned independent parallel “backup campaign”).

Beyond the first large‐scale supply, the time to reach output milestones (such as the billionth dose) is a function of the resources committed (e.g., square meter of production facility) and the output in doses per square meter per day. The latter is a function of volumetric productivity and cycle time. The perfusion‐based upstream process for ChAdOx1 nCoV‐19 production described here boosts volumetric productivity by approximately fourfold compared with the current process (reaching approximately 8 × 10^14^ VP of purified DS per liter of bioreactor culture, sufficient for >10,000 doses of drug product). To our knowledge this is the first demonstration that perfusion can improve productivity of a species E adenoviral vector or a simian adenoviral vector. Our results are favorably comparable to those reported with perfusion‐based processes for the production of species B, C, and D vectors, including in PER.C6 cells (Galvez et al., 2012; Luitjens & Lewis, 2018; Luitjens & Van Herk, 2016).

Our “design of experiments” (DoE) approach on the Ambr250 HT perfusion system identified early perfusion start and intensified perfusion after infection as factors that improved volumetric productivity. We included early perfusion start but not intensified perfusion after infection in the design of a new perfusion‐based upstream process for testing in a 3‐L system. In this work we demonstrated reproducible performance of the process, using a constant rate of perfusion (1 vessel volume/day) from 48 h before infection to harvest at 48 h after infection. Our rationale for not intensifying perfusion was that facilities manufacturing ChAdOx1 nCoV‐19 are already capable of handling 1 vessel volume/day of medium (as a requirement of the current process), but may not be capable of handling intensification to 2 vessel volumes/day. The large improvement in upstream productivity is compatible with the existing downstream process. We demonstrated product recovery (approximately 50%) and product quality in line with expectations.

The work described here was facilitated by the fact that adenovirus production remains a brief batch process, rather than a long continuous process (in which issues such as filter fouling may be more problematic). The work was also facilitated by the use of novel small‐scale model systems. Previous small‐scale models of perfusion have relied on the discontinuous medium exchange by intermittent centrifugation and aspiration of the spent medium. This provides an imperfect model of metabolite concentrations or shear forces in cultures undergoing continuous medium exchange through a filter. This may be the first published report of multiparallel optimization of a true perfusion process (“DoE” in a total of 25 reactors, enabled by the first published use of the Ambr250 HT perfusion system). It may also be the first published report of use of the XCell Lab ATF controller and ATF1 filter system (enabling modeling of ATF filter dynamics in a 2000‐L process at 3 L laboratory scale). Extension of this work to confirm process scalability beyond benchtop vessels would now be desirable.

Overall, this work suggests the suitability of the adenovirus‐vectored vaccine platform for rapid emergency response, and the feasibility of considerable improvement upon the productivity of a manufacturing process that already, at the time of writing, is estimated to have delivered more than three billion doses (Global Commission for Post‐pandemic Policy, 2022). There remains scope for further improvement in the time to both trial‐scale and large‐scale supply, and the facility footprint required to achieve a given level of output. The methods described here, however, are likely to be sufficient to reduce differences in manufacturing speed and facility output versus other platforms, including mRNA, to a level at which other factors (such as safety, tolerability, efficacy, and stability) become more important in considering the ongoing roles of different platforms in emergency response. Public health benefit is likely to be maximized by the availability of diverse vaccines, including adenoviruses, and there may be valid scientific reasons for the selection of different vaccines in different contexts.

## 4 METHODS

### 4.1 Cells and vaccine

All experiments used a previously described research cell bank of T‐REx‐293 human embryonic kidney (HEK) cells stably expressing the tetracycline repressor protein (ThermoFisher) (Fedosyuk et al., 2019). Cells were adapted to antibiotic‐free and serum‐free BalanCD HEK293 medium (Irvine) and maintained as previously described (Joe et al., 2021). Cells were expanded in medium supplemented with 4 mM l‐alanyl‐l‐glutamine (Gibco) in 125 mL and 1 L Erlenmeyer flasks (Corning) and 5 L Optimum Growth (Thomson) flasks using humidified shaking incubators (Kühner) at 37°C, 8% CO_2_ and 130 rpm with a 25‐mm orbit. Cells were subcultured every 3–4 days to maintain viable cell density between 0.5 × 10^6^ and 3 × 10^6^ cells/mL at a maximum fill volume of 20%. Viable cell density was measured daily using a NucleoCounter NC‐202 (ChemoMetec).

ChAdOx1 nCoV‐19 was derived as previously reported (van Doremalen et al., 2020). A seed stock was prepared for the present study using our previously described fed‐batch manufacturing process (Joe et al., 2021). Except where otherwise stated, seed was used to infect cells at an MOI of 5 IU/cell.

### 4.2 Product quantification

ChAdOx1 nCoV‐19 was quantified as previously described, using qPCR and ultraviolet spectrophotometry assays for VPs and a cell‐based immunostaining assay for IUs (Fedosyuk et al., 2019).

ChAdOx1 nCoV‐19 in cell culture samples was quantified using qPCR and infectivity assays following cell lysis (using the method described below for virus harvest). ChAdOx1 nCoV‐19 in ion‐exchange eluate samples and in purified product was quantified by ultraviolet spectrophotometry before addition of polysorbate 80.

For the avoidance of doubt, throughout this manuscript the term “viral particle,” abbreviated “VP,” is used to refer to genome‐containing VPs. Empty viral capsids present in the samples are not detected by qPCR. Nor does the qPCR detect unencapsidated viral genomes (these are destroyed by the nuclease treatment which precedes lysis of the viral capsids during qPCR sample preparation).

Each sample was assayed in triplicate wells on duplicate or triplicate plates (unless otherwise stated), each with standard curve samples (10^6^–10^10^ genome copies spaced at 10‐fold intervals) and two internal controls. These comprised research‐grade ChAdOx1 nCoV‐19 purified by CsCl gradient ultracentrifugation and quantified by spectrophotometry, and clinical‐grade ChAdOx1 nCoV‐19 quantified by a GMP‐qualified external qPCR assay (Advent, IRBM SpA). The acceptance criteria for qPCR values were: accuracy of internal controls within 15%, efficiency of 90%–110%, and standard curve linearity (*R* ^2^) of at least 0.99. Typical standard errors of the between‐plate mean (calculated from individual plate results) were c. 15% of the mean and no higher than 30%.

Quantification of VP by UV spectrophotometry is based upon absorbance at 260 nm, which is more strongly influenced by DNA (characteristic *A*_260_:*A*_280_ ratio = 1.8) than protein (characteristic *A*_260_:*A*_280_ ratio = 0.6). Empty viral capsids present at up to 25% of total capsids (as seen in Figure 5d) will thus cause UV spectrophotometric measurements to overestimate genome‐containing virus particles by ≤15%.

### 4.3 Rapid virus seed production

An Erlenmeyer flask with a culture volume of 30 mL and a viable cell density of 0.8 × 10^6^ cells/mL received ChAdOx1 nCoV‐19 at an MOI of 6 VP/cell (as determined by qPCR). This qPCR value corresponds to approximately 0.1 IU/cell with a plausible range of 0.025–0.2 IU/cell assuming a typical particle:infectivity ratio for ChAdOx1 nCoV‐19. The cells were harvested and lysed 6 days later. Virus was partially purified using depth filtration and AEX chromatography with syringes and small‐scale filters (C0SP 23 cm^2^ [Merck] and Sartobind Q Nano 3 mL [Sartorius]). Buffer compositions and other parameters were as previously described, but scaled proportionately to the product and filter sizes (Joe et al., 2021). Bioreactors were seeded with approximately 0.5 × 10^6^ cells/mL in 2.4 L (80% of the 3 L maximum working volume), and infected 28–30 h later at an MOI of 6 VP/cell and viable cell density of approximately 0.8 × 10^6^ cells/mL. Cell cultures were fed with BalanCD HEK293 feed 5% v/v at 48 h and 96 h after infection (±4 h). As previously described for low‐MOI DS production, temperature was reduced within 4 h of the second feed. Cultures were harvested approximately 140 h after infection and virus was partially purified as described above.

### 4.4 High‐throughput perfusion studies

#### 4.4.1 Multiparallel bioreactors

For high‐throughput screening of process conditions, the multiparallel bioreactor and perfusion system Ambr250 High‐Throughput Perfusion (Ambr250 HT perfusion; Sartorius) was used with 0.2 µm hollow‐fiber filters in ATF mode. The total reactor volume was 210 mL/vessel and the stirring power input was 40 W/m^3^. Temperature was controlled at 37°C, dissolved oxygen was controlled at 55% by sparging with air and/or O_2_, and pH was controlled at 7.25 (dead‐band, 0.05) by sparging with CO_2_ mix or addition of 1 M sodium bicarbonate. Antifoam C emulsion 30% (Sigma‐Aldrich) was diluted to 2% and automatically added as required via the Ambr250 integrated foam sensor and liquid handler. Each Ambr250 vessel was filled with prewarmed BalanCD HEK293 medium and seeded with 0.5 × 10^6^ viable T‐REx‐293 cells/mL. After 3 days, cells were bled and replenished to reactor working volume containing 1 × 10^6^ viable cells/mL (thus modeling preproduction “*n* − 1” cell seed culture expansion in a stirred‐tank bioreactor). Nutrient supplementation used 5% BalanCD HEK293 feed, as described below.

Viable cell density was measured daily using a Cedex HiRes Analyzer (Roche). Metabolites were automatically analyzed daily via the integrated BioProfile FLEX2 device (Nova Biomedical). Samples for adenoviral vector quantification by qPCR (see below) were taken 44 and 48 h after infection. For each time point, VP counts were calculated as the mean of triplicate samples, each analyzed in triplicate wells on triplicate plates. The mean of the 44‐ and 48‐h values was used for analysis.

#### 4.4.2 Experimental design

A “DoE” approach was used to determine the influence of three factors on productivity: PST, at a rate of one reactor volume per day; high or low VCDI; and duration of intensified perfusion (at a rate of two reactor volumes per day, starting at infection). When the duration of intensified perfusion was zero, perfusion continued at one reactor volume per day after infection. Two successive full‐factorial experiments each comprised two levels of these three factors (2 × 2 × 2), in eight single bioreactors. Each experiment also included one “center‐point” condition (in triplicate bioreactors) and one “simplified center‐point‐like” condition (singly or in duplicate), for a total of 12 parallel bioreactors in the first experiment and 13 in the second (Supporting Information Table 1).

The first experiment was designed to target a VCDI of approximately 4 × 10^6^ or 12 × 10^6^ cells/mL with a center point of c. 8 × 10^6^ cells/mL; a PST of 12 or 48 h before infection, with a center point of 24 h; and an IPD of 0 or 48 h after infection, with a center point of 24 h. In the second experiment, the design was adjusted to target viable cell densities at infection of approximately 6 × 10^6^ or 18 × 10^6^ cells/mL with a center point of c. 12 × 10^6^ cells/mL; the PST was set to 48 or 72 h after cell density adjustment by reactor bleed, with a center point of 60 h; and the duration of intensified perfusion was as in the first experiment.

The “simplified center‐point‐like” condition used center‐point VCDI, early PST, and an IPD of 0 h (i.e., no intensification of perfusion; continuous perfusion at one reactor volume per day). This condition was designed to balance high productivity (via early perfusion start and intermediate cell density) with the suitability of the process for multiple facilities (avoiding the facility‐fit and medium‐supply challenges that might arise from handling 2 vessel volumes of medium per day at a large scale).

The difference in triggering of PST between the two experiments provided a range of viable cell densities at the time perfusion was started, with higher cell densities in the second experiment than the first. The experiments were performed successively, enabling observations made during the first experiment to inform the design of the second. For the second experiment the maximum fill volume during the last shake flask cultivation step was reduced to 10% (to reduce aggregation of the cells before inoculation of the Ambr250 HT perfusion bioreactor vessels). In addition, nutrient supplementation was increased by the addition of 5% feed solution at the bioreactor bleed 3 days after inoculation (to support the higher cell densities in the second experiment).

Two response variables were defined: volumetric productivity (VP/mL), and cell‐specific productivity (VP/cell). Cell‐specific productivity values were based on the peak viable cell density for the reactor recorded by the Cedex Analyzer.

#### 4.4.3 Statistical analyses

DoE analyses were performed using MODDE version 13 software (Sartorius). The main DoE analysis combined the two experiments using a dummy variable denoting each experiment (EXP). To combine the experiments, the PST factor was replaced with a continuous variable describing the PSVCD. When necessary, PSVCD was calculated by extrapolation or interpolation from the nearest available cell count and population doubling time (in all cases, within 24 h of perfusion start). One center‐point condition reactor from experiment 2 was excluded from all analyses owing to microbial contamination. The response variables were normalized with a log_10_ transformation. In the main analysis, a model including the terms EXP, PSVCD, VCDI, and IPD was fit by partial least‐squares regression. Statistical significance of parameter coefficients was assessed using *t* tests (with a null hypothesis that parameter coefficients were zero) and values of *r* ^2^ and *q* ^2^ were calculated using functions in MODDE as defined in the software user guide.

Eight sensitivity analyses were performed to evaluate the impact on the overall conclusions of the following changes from the above analysis. First, the PST factor was replaced with perfusion duration before infection instead of PSVCD. Second, raw response data were used instead of log_10_‐transformed data. Third and fourth, each experiment was analyzed individually instead of in combination. Fifth, the experiments were pooled without using the EXP dummy variable. Sixth, three bioreactors with simplified center‐point‐like conditions that did not fit the full‐factorial design were excluded. Seventh, two bioreactors were excluded owing to initial low cell density likely resulting from error during setup (one center‐point reactor in the first experiment and one simplified center‐point‐like reactor in the second experiment). Finally, the model was fitted using multiple linear regression instead of partial least‐squares regression.

## 5 LITER PRODUCTION BIOREACTORS

We designed perfusion filter dimension and flow parameters, using XCell Lab technology (Repligen) to provide the most accurate possible model of 2000 L production at benchtop scale. A 0.022‐m^2^ filter (ATF1, Repligen) used with ATF at 5 L/m^2^/min to exchange medium in a 3‐L working‐volume vessel at 1 vessel volume/day (0.1 L/m^2^/min) provides shear rate (1516/s) and other fluid dynamic parameters similar to those of an 11‐m^2^ filter (ATF10, Repligen) used for perfusion of a 2000‐L bioreactor of circa 1500 L working volume under recommended conditions. These flow rates fall comfortably within commonly used operating ranges.

BioBLU 3c single‐use bioreactors with an open pipe, a pitched‐blade impeller, and an optical pH port were used in a BioFLo320 parallel bioreactor system (Eppendorf) with integrated intelligent sensor management (Mettler Toledo). The submerged addition line was welded to the bioreactor connection line of a single‐use hollow‐fiber ATF filter and pump device with a 0.22‐µm pore size, a 1‐mm lumen, and a 218‐cm^2^ nominal surface area (XCell ATF1, Repligen). ATF was set to 0.11 L/min (5 L/m^2^/min) using an XCell Lab Controller (Repligen). The desired rate of medium exchange was achieved by controlling the flow rate of the permeate (spent medium) and adding a new medium, both via the BioFlo320 integrated pumps (after calibration). The new medium feed pump was controlled by a gravimetric feedback loop programmed to maintain constant reactor weight.

Bioreactor growth medium was BalanCD HEK293 supplemented with 4 mM l‐alanyl‐l‐glutamine, 0.01% antifoam C emulsion (Sigma‐Aldrich), and 10%–20% BalanCD HEK293 feed (Irvine). Bioreactors were stirred at 160–180 rpm (26–33 W/m^3^) and maintained at 37°C using a heating jacket. Dissolved oxygen was controlled at >55% by sparging with air and/or O_2_ via a cascading control loop. The pH was controlled at 7.30 (dead‐band, 0.1) by sparging with CO_2_ or the addition of 7.5% sodium bicarbonate. Initial T‐REx‐293 cell density was 0.5–2 × 10^6^ viable cells/mL in a working volume of 3 L. After infection with ChAdOx1 nCoV‐19, samples were taken daily for adenoviral vector quantification and biochemical analysis using a Stat Profile Prime analyzer (Nova Biomedical).

### 5.1 Harvest and concentration

Cells were lysed 42–48 h after infection with ChAdOx1 nCoV‐19 by addition of 1/9 culture volume of 10% v/v polysorbate 20, 50% w/v sucrose, and 20 mM MgCl_2_ in 500 mM Tris–HCl pH 8.0, 15 min after addition of benzonase (Merck Millipore) to a final concentration of 100 U/mL. Dissolved oxygen and pH were uncontrolled during lysis, but stirring and temperature control continued. After 2 h, the lysate was clarified over 270 cm^2^ Millistak+ HC Pro C0SP depth filters coupled with Opticap XL150 0.2 µm sterile filters (Merck Millipore) at a flow rate of 3.3 L/min/m^2^ as previously described (Fedosyuk et al., 2019). Turbidity was measured using a TN‐100 waterproof turbidimeter (Thermo Scientific Eutech).

In one experiment, the clarified lysate was concentrated by TFF at 5 L/min/m^2^ using a KR2i KrosFlo (Repligen) with Pellicon 2 mini cassettes (300 kDa, 0.1 m^2^, BioMax polyethersulfone membrane with C‐screen; Merck), as previously described (Fedosyuk et al., 2019), but with the addition of a KRJr permeate control pump (Repligen) set to 0.66 L/min/m^2^. Feed pressure was controlled at 0.7 bar by an automated backpressure valve on the retentate outflow and the diafiltration buffer feed rate was matched to the permeate flow rate by controlling the weight of the process reservoir. After twofold concentration, the retentate underwent diafiltration with one diavolume of ion‐exchange wash buffer (222 mM NaCl, 1 mM MgCl_2_, and 5% w/v sucrose in 50 mM Tris–HCl, pH 8.0, with a conductivity of 23–24 mS/cm).

### 5.2 Purification

After clarification, lysates were purified by ion‐exchange chromatography. AEX was preceded in one case (experiment CJ74) by limited TFF as described above, and in others by dilution of lysate 1:3 with wash buffer (as above). Before chromatography, the conductivity of the lysate was adjusted to 23–24 mS/cm using 5 M NaCl. Fresh single‐use SartobindQ capsules with 150 mL bed volume and 8 mm bed height were used in a peristaltic pump‐driven rig incorporating single‐use ultraviolet absorbance, conductivity, and pressure sensors (Pendotech), as previously described (Fedosyuk et al., 2019). Capsules were washed, equilibrated, loaded, and eluted in accordance with the manufacturer's instructions. Briefly, the membrane was sanitized with 30 membrane volumes (MVs) of 1 M NaOH at 1 MV/min, activated with 10 MV of 1 M NaCl at 2 MV/min, and equilibrated with 20 MV of ion‐exchange wash buffer (see above) at 2 MV/min. Samples were applied at 2.5–3.0 MV/min, washed as per the equilibration step, and eluted with 5 MV of 444 mM NaCl, 1 mM MgCl_2_, and 5% w/v sucrose in 50 mM Tris–HCl, pH 8.0, with a conductivity of 39–40 mS/cm, at 2 MV/min. Eluate was collected into a reservoir containing a “cushion” of charge buffer (35 mM NaCl, 10 mM histidine, 1 mM MgCl_2_, 0.1 mM EDTA, 7.5% w/v sucrose, 0.5% v/v ethanol, and pH 6.6).

After ion‐exchange chromatography, the eluate buffer was exchanged with 6 diavolumes of 10 mM histidine, 7.5% sucrose, 35 mM sodium chloride, 1 mM magnesium chloride, 0.1 mM ethylenediaminetetraacetic acid, 0.5% (v/v) ethanol, and pH 6.6 (i.e., formulation buffer A438 without polysorbate 80) (Evans et al., 2005). This final TFF step was performed using Pellicon 2 mini cassettes (as above) with a feed flow rate of 5 L/min/m^2^, a feed pressure of 0.7 bar, and a permeate flow rate of 0.66 L/min/m^2^ (matched by the rate of addition of fresh diafiltration buffer via an automated auxiliary pump). Finally, polysorbate 80 was added to a final concentration of 0.1% v/v and the product was passed through 0.2 µm filters (Nalgene).

### 5.3 Analysis of residuals and electron microscopy

Residual HCP was quantified using the HEK293 HCP ELISA kit (Cygnus Technologies) according to the manufacturer's instructions. Residual host‐cell DNA was quantified using the previously reported qPCR method, with a lower limit of quantification of 100 pg/mL for intact HEK293 cell DNA (Fedosyuk et al., 2019). Polyacrylamide gel electrophoresis was conducted using standard molecular biology laboratory techniques.

Samples were diluted to 3–5 × 10^11^ VP/mL in water as required and applied to freshly glow‐discharged, carbon‐filmed, 300‐mesh copper grids (TAAB Laboratories). Grids were incubated at room temperature for 2 min, blotted, immediately transferred to a 20‐µL droplet of 2% uranyl acetate for 10 s, then blotted and air dried. Images were acquired at 120 keV on a Tecnai 12 transmission electron microscope (ThermoFisher) equipped with a OneView camera (Gatan).

### 5.4 Additional analysis

Graphs other than those produced using MODDE software (see above) were prepared using Prism 9.0 (GraphPad Software).

## AUTHOR CONTRIBUTIONS

*Conceptualization*: Alexander D. Douglasimitrios Doultsinos and Carina C. D. Joe. *Methodology*: Alexander D. Douglasimitrios Doultsinos, Carina C. D. Joe, and Julia Niemann. *Investigation*: All. *Funding acquisition, project administration, and supervision*: Alexander D. Douglasimitrios Doultsinos and Julia Niemann. *Writing original draft*: Alexander D. Douglasimitrios Doultsinos, Carina C. D. Joe, Steffi Scholze, and Julia Niemann. *Reviewing and editing*: All.

## CONFLICT OF INTEREST STATEMENT

Carina C. D. Joe, Yuanyuan Li, Adam Berg, Rameswara R. Segireddy, and Alexander D. Douglas are named inventors or contributors to intellectual property assigned to Oxford University Innovation relating to the ChAdOx1 nCoV‐19 vaccine and/or manufacturing process, and receive a proportion of proceeds from out‐licensing of this intellectual property. Alexander D. Douglas has received consultancy income from AstraZeneca.

## Supporting information

Supporting information.

## Data Availability

The data that support the findings of this study are available from the corresponding author upon reasonable request. All data needed to evaluate the conclusions in the paper are present in the paper and/or the Supporting Information. Raw data are available from the corresponding author upon reasonable request.
