# Supplementary material for: Accelerated and intensified manufacturing of an adenovirus‐vectored vaccine to enable rapid outbreak response
Source: Biotechnol Bioeng. 2023 Sep 25;121(1):176–91. doi: 10.1002/bit.28553 (PMC10932548; doi:10.1002/bit.28553)
Supplement: Supplementary file 1 — Supporting information. [file BIT-121-176-s001.docx]

# Supplement

## Supplementary Tables

#### Supplementary Table 1 Variable and response levels in each bioreactor.

| Bioreactor | Variables | | | |  | Responses | |
| --- | --- | --- | --- | --- | --- | --- | --- |
|  | **PDBI (hours)** | **PSVCD (10^6^ cells/mL)** | **VCDI (10^6^ cells/mL)** | **IPD (hours)** |  | **Volumetric productivity**  **(VP/mL)** | **Cell-specific productivity (VP/cell)^a^** |
| **Experiment 1** | | |  |  |  |  |  |
| 1 | 12 | 3.74 | 4.57 | 0 |  | 8.9 × 10^11^ | 1.3 × 10^5^ |
| 2 | 48 | 1.25 | 3.67 | 0 |  | 6.5 × 10^11^ | 9.8 × 10^4^ |
| 3 | 12 | 6.58 | 7.39 | 0 |  | 6.2 × 10^11^ | 6.0 × 10^4^ |
| 4 | 48 | 3.40 | 10.44 | 0 |  | 8.7 × 10^11^ | 6.0 × 10^4^ |
| 5 | 12 | 4.02 | 4.71 | 48 |  | 8.6 × 10^11^ | 1.1 × 10^5^ |
| 6 | 48 | 1.57 | 5.09 | 48 |  | 7.8 × 10^11^ | 9.4 × 10^4^ |
| 7 | 12 | 6.16 | 7.10 | 48 |  | 6.6 × 10^11^ | 7.9 × 10^4^ |
| 8 | 48 | 3.43 | 11.19 | 48 |  | 1.3 × 10^12^ | 8.3 × 10^4^ |
| 9^b^ | 30 | 2.99 | 5.78 | 24 |  | 1.3 × 10^12^ | 1.1 × 10^5^ |
| 10^b,d^ | 30 | 3.52 | 5.24 | 24 |  | 8.5 × 10^11^ | 9.5 × 10^4^ |
| 11^b^ | 30 | 3.42 | 5.59 | 24 |  | 1.2 × 10^12^ | 9.5 × 10^4^ |
| 12^c^ | 48 | 2.83 | 6.40 | 0 |  | 8.7 × 10^11^ | 9.1 × 10^4^ |
| **Experiment 2** | | |  |  |  |  |  |
| 1 | 24 | 4.39 | 6.93 | 0 |  | 5.0 × 10^11^ | 5.1 × 10^4^ |
| 2 | 0 | 5.95 | 5.95 | 0 |  | 4.2 × 10^11^ | 4.7 × 10^4^ |
| 3 | 72 | 3.76 | 17.15 | 0 |  | 5.0 × 10^11^ | 2.7 × 10^4^ |
| 4 | 48 | 6.30 | 16.09 | 0 |  | 4.6 × 10^11^ | 2.5 × 10^4^ |
| 5 | 24 | 4.25 | 6.71 | 48 |  | 7.0 × 10^11^ | 7.9 × 10^4^ |
| 6 | 0 | 6.13 | 6.13 | 48 |  | 6.5 × 10^11^ | 7.0 × 10^4^ |
| 7 | 72 | 3.68 | 16.90 | 48 |  | 8.0 × 10^11^ | 4.4 × 10^4^ |
| 8 | 48 | 6.79 | 17.50 | 48 |  | 8.2 × 10^11^ | 3.9 × 10^4^ |
| 9^b,e^ | 36 | 4.88 | 9.79 | 24 |  | 6.2 × 10^10^ | 5.8 × 10^3^ |
| 10^b^ | 36 | 5.47 | 11.03 | 24 |  | 5.8 × 10^11^ | 4.0 × 10^4^ |
| 11^b^ | 36 | 5.53 | 11.43 | 24 |  | 6.9 × 10^11^ | 5.0 × 10^4^ |
| 12^c^ | 48 | 4.23 | 10.96 | 0 |  | 9.9 × 10^11^ | 7.5 × 10^4^ |
| 13^c,d^ | 48 | 2.28 | 6.68 | 0 |  | 4.1 × 10^11^ | 5.5 × 10^4^ |

^a^ Based on peak viable cell density (not shown).

^b^ Centre-point condition.

^c^ Simplified centre-point-like condition.

^d^ Outlier with low cell density.

^e^ Contaminated and excluded from analyses.

IPD, intensified perfusion duration; PDBI, perfusion duration before infection; PSVCD, perfusion-start viable cell density; VCDI, viable cell density at infection; VP, viral particles.

#### Supplementary Table 2 Step recovery and filter loading in three independent downstream process runs

|  | EXPERIMENT | | | |
| --- | --- | --- | --- | --- |
| **PARAMETER** | **CJ74** | **CJ79** | **CJ87** | **Mean** |
| **Step recovery by qPCR** |  |  |  |  |
| Clarification​ | 73​% | 63​% | 63%​ | 66% |
| Pre-AEX chromatography TFF​* | 95​% | N/A​ | N/A​ | N/A |
| AEX​ chromatography | 89​% | 100%​ | 83%​ | 91% |
| Formulation TFF​ | 98​% | 82​% | 88​% | 89% |
| 0.2 μm filtration​ | 92​% | 92%​ | 100​% | 95% |
| Cumulative recovery (DS as % of upstream) | 56% | 48% | 46% | 50% |
| **Filter loadings** |  |  |  |  |
| Clarification (cells/m^2^)​ | 1.1 × 10^12​^ | 1.1 × 10^12^​ | 1.1 × 10^12^​ |  |
| AEX chromatography (VP per millilitre of membrane)​ | 6.3 × 10^12^​ | 8.2 × 10^12^​ | 1.2 × 10^13^​ |  |

*Additional step used in CJ74 only.

AEX, anion-exchange; DS, drug substance; N/A, not applicable; qPCR, quantitative PCR; TFF, tangential-flow filtration; VP, viral particles.

## Supplementary Figures

#### Supplementary Figure 1. Cell growth and productivity in multi-parallel scaled-down bioreactor experiments.


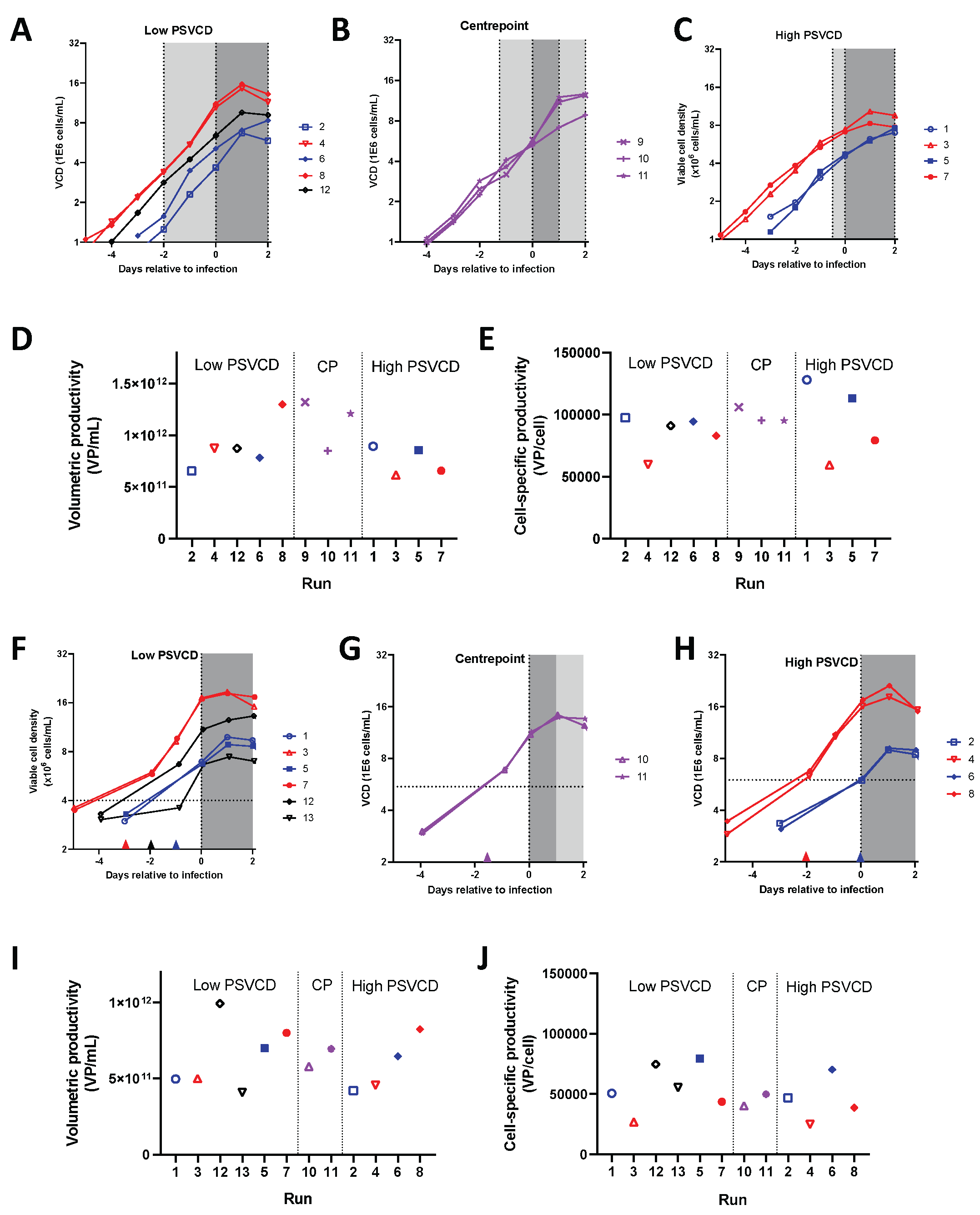


*Data in panels A–E is from Experiment 1; data in panels F–J is from experiment 2. Panels A–C and F–H show cell growth in each reactor; D–E and I–J show response variables.*

*Filled symbols indicate intensified perfusion for 48 h after infection; open symbols indicate no intensified perfusion (NB, centre point reactors used intensified perfusion for 24 h after infection). Blue indicates low and red indicates high viable cell density at infection; purple indicates centre point and black indicates simplified centre-point like viable cell density at infection. Light grey shading (A–C; G) or arrowheads (F–H) indicate non-intensified perfusion. Dark grey shading indicates intensified perfusion in some reactors.*

*CP, centre point; PSVCD, perfusion-start viable cell density; VCD, viable cell density; VP, viral particles.*

#### Supplementary Figure 2. Sensitivity analyses of multi-parallel scaled-down bioreactor experiments for (A) cell-specific productivity and (B) volumetric productivity.


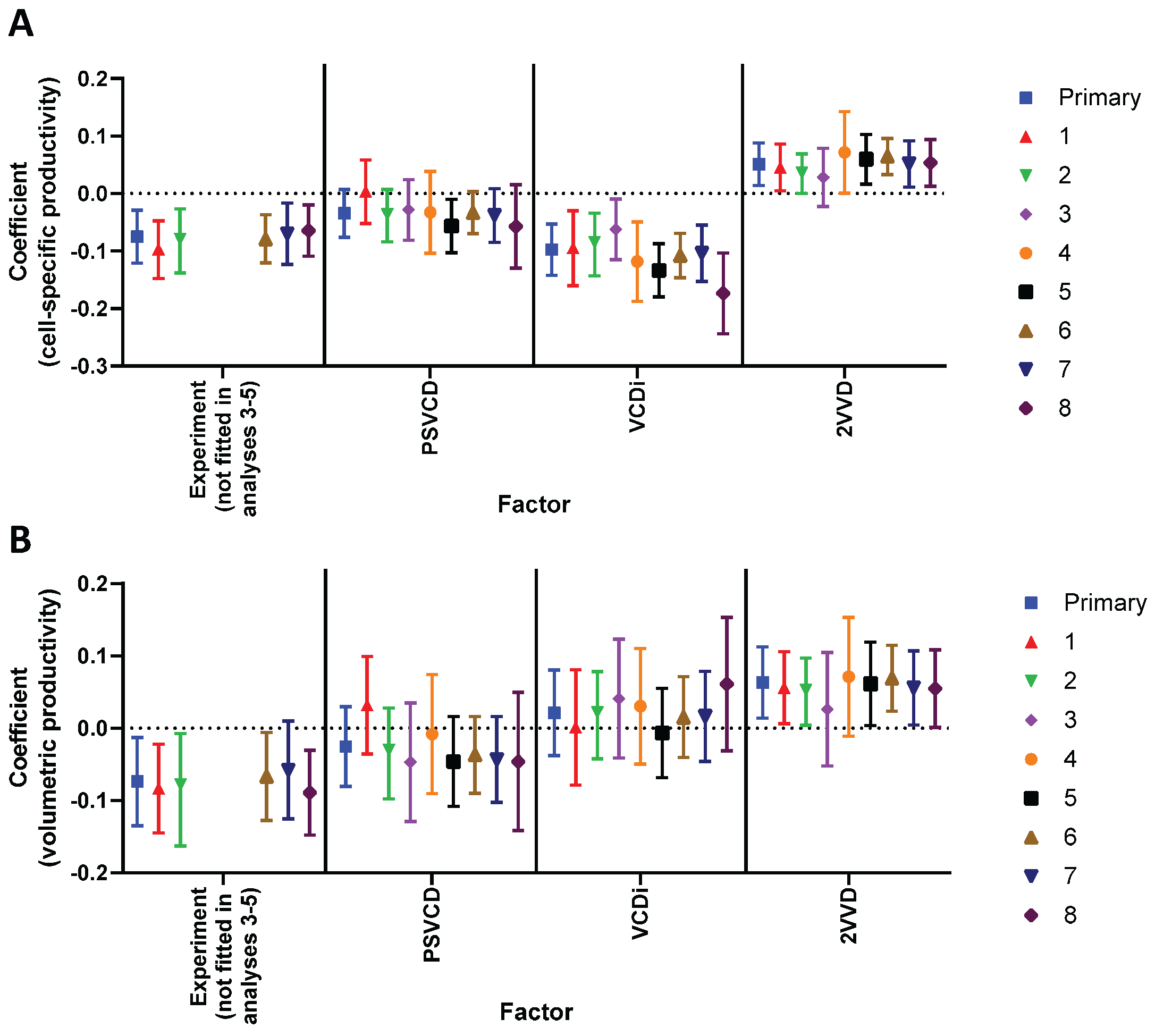


Legend: 1, PST factor replaced with perfusion duration before infection (PDBI) instead of PSVCT; 2, raw response data instead of log_10_-transformed data; 3–4, experiments analysed individually instead of in combination; 5, pooling of experiments without the EXP dummy variable; 6, exclusion of three bioreactors with simplified centre-point-like conditions that did not fit the full-factorial design; 7, exclusion of two bioreactors due to initial low cell density likely resulting from error during set-up (one centre-point reactor in the first experiment and one simplified centre-point-like reactor in the second experiment); 8, model fit using multiple linear regression instead of partial least-squares regression.

PSVCD, perfusion-start viable cell density; VCDI, viable cell density at infection, 2VVD, intensified perfusion with 2 vessel volumes per day.
